# SRM dataset of the proteome of inactivated iron-sulfur cluster biogenesis regulator SufR in *Synechocystis* sp. PCC 6803

**DOI:** 10.1016/j.dib.2017.03.012

**Published:** 2017-03-11

**Authors:** Linda Vuorijoki, Pauli Kallio, Eva-Mari Aro

**Affiliations:** Molecular Plant Biology, Department of Biochemistry, University of Turku, FI-20014, Turku, Finland

## Abstract

This article contains SRM proteomics data related to the research article entitled”Inactivation of iron-sulfur cluster biogenesis regulator SufR in *Synechocystis* sp. PCC 6803 induces unique iron-dependent protein-level responses” (L. Vuorijoki, A. Tiwari, P. Kallio, E.M. Aro, 2017) [1]. The data described here provide comprehensive information on the applied SRM assays, together with the results of quantifying 94 *Synechocystis* sp. PCC 6803 proteins. The data has been deposited in Panorama public (https://panoramaweb.org/labkey/SufR) and in PASSEL under the PASS00765 identifier (http://www.peptideatlas.org/PASS/PASS00765).

**Specifications Table**

**Table:** 

Subject area	Biochemistry
More specific subject area	SRM proteomics
Type of data	Text file, Excel file
How data was acquired	TSQ Vantage (Thermo Scientific)
Data format	Raw, analyzed, table
Experimental factors	Proteins were extracted from *Synechocystis* wild type and mutant strains grown under iron sufficient and depleted conditions, and subjected to SRM analysis.
Experimental features	SRM proteomics was applied to the *Synechocystis* cells grown under different iron concentrations. The proteomics data was analyzed and processed with Skyline software and MSstats statistical tool.
Data source location	University of Turku, Turku, Finland
Data accessibility	The data described in this article can be accessed in Panorama Public [2] (https://panoramaweb.org/labkey/SufR) and PASSEL [3] with an identifier PASS00765 (http://www.peptideatlas.org/PASS/PASS00765).

**Value of the data**

• The dataset contains detailed information about SRM assays for quantification of 94 *Synechocystis* sp. PCC 6803 proteins.
• The targets cover various key metabolic entities in *Synechocystis*, with the emphasis on Fe-associated functions.
• With the dataset, the publicly available SRM assay library specific for *Synechocystis* is more inclusive.
• The data can be used and supplemented in further SRM assays.
• The data show the effect of *sufR* deletion in *Synechocystis* on set of pre-selected proteins under varying iron concentrations [1].

## 1 Data

The data contains information on SRM assays for 94 pre-selected *Synechocystis* sp. PCC 6803 proteins analyzed from unfractionated samples. Represented are i) the SRM results in a complete, processed Skyline document deposited in Panorama Public repository, ii) the raw data and transition lists deposited in the Peptide Atlas SRM Experiment Library (PASSEL) as well as iii) indexed peptide retention times, iv) MSstats input file and v) statistical information on the changes in expression levels described with this article. The proteins subjected to SRM analysis, were extracted from wild type and *sufR* deletion mutant strains grown under iron sufficient and deprived conditions.

## 2 Experimental design, materials and methods

### 2.1 Growth conditions

*Synechocystis* sp. PCC 6803 *sufR* deletion mutant and the wild type strains were cultivated under photoautotrophic conditions in AlgaeTron AG 230 growth chamber (Photon Systems Instruments, Drásov, Czech Republic), under controlled conditions, with 1% CO_2_ (v/v). The temperature was set to +30 °C and the light intensity to 50 µmol photons m^−2^ s^−1^. The cells were grown in BG-11 medium [4], buffered with 20 mM TES-KOH (pH 8.0) in Erlenmeyer culture flasks on a rotary shaker (120 rpm). The precultures (40 ml BG-11 in 100 ml flasks) were grown under standard BG-11 media until mid-logarithmic phase, harvested by centrifugation and washed either one or three times in standard or iron-depleted BG-11 media, respectively. The precultures were used to inoculate the main cultures (40 ml BG-11 in 100 ml flasks) to the starting OD_750nm_ of 0.1. The cell density was estimated by measuring the optical density at 750 nm (OD_750nm_) with a Genesys 10 S UV–vis spectrophotometer (Thermo Scientific). The cells were harvested from both iron sufficient and deprived conditions at OD_750nm_ 1.0, and after 12 days under iron deprivation as described in [5]. In order to remove all traces of iron from the culture flask used for iron depleted growth, the glassware was treated with 10% HCl, and rinsed thoroughly with MQ-water prior autoclaving.

### 2.2 Sample preparation

Cells for the proteomics analysis were collected by centrifugation and washed twice with 50 mM TES-KOH buffer, pH 8.0. The cell pellet was suspended in protein extraction buffer [0.1% (w/v) RapiGest SF in 8 M urea solubilized in 0.1 M NH_4_HCO_3_ supplemented with 200 µM PMSF] with equal volume of acid-washed glass beads (150–212 µm, Sigma). The cells were broken by a bead beater and the insoluble fraction and glass beads were removed by centrifugation. The proteins were reduced with dithiotreitol and alkylated with iodoacetamide. The protein extracts were precipitated with 1:5 v/v of 50% acetone/ 50% ethanol o/n at −20 °C. The pellet was solubilized by trypsin digestion (1:100 trypsin:protein ratio) in 50 mM NH_4_HCO_3_ and 5% acetonitrile (ACN) buffer for 4–5 h at +37 °C shaking. Digestion was continued for an additional 15–16 h following a second addition of trypsin in the same ratio. The digestion was stopped by adding formic acid (FA) (Sigma) to a final concentration of 0.5–1% to lower the pH below 2. The digestion mixture was incubated for 30 min at +37 °C at 130 rpm and centrifuged. To desalt the samples, solid phase extraction of the peptide mixture was performed with 4 mm/1 ml extraction disk cartridge (Empore™ C18-SD, 3 M™) according to manufacturer׳s protocol. The eluted peptide samples were vacuum-dried (Savant SPD1010, SpeedVac Concentrator, Thermo Scientific) and solubilized in 0,1% FA and 2% ACN and stored at −80 °C prior to MS analysis.

### 2.3 SRM analysis and data-processing

The SRM assays were performed on a TSQ Vantage QQQ mass spectrometer (Thermo Scientific) equipped with a nanoelectrospray ionization source. The desalted peptides were separated on a nanoflow HPLC system (EasyNanoLC 1000; Thermo Scientific). The amount of each injected unfractionated biological triplicate was 150 ng. A 60 min non-linear gradient (5–20% B in 35 min; 20–35% B in 50 min; B = ACN:water, 98:5) was applied at a 300 nl/ min flow rate. Once the peptides were eluted and ionized, they were analyzed in the QQQ-MS, operated in the SRM mode, as described in [5]. To maintain high sensitivity of the SRM measurements, the selected targets were divided to three different transition lists and scheduled assays with a 5 min retention time window for each peptide was applied, resulting in a cycle time of 2.5 s and a dwell time of >20 ms. The protein targets and the respective SRM assay parameters were selected from the public dataset, available in Panorama Public (https://panoramaweb.org/labkey/Vuorijoki_et_al_2015.url) [5]. For the quantification of six proteins (encoded by *sll0542, sll1031, sll1525, slr1963, slr2067, slr2136*), a different set of 2–3 proteotypic peptides were selected, due to more stable signals between the replicates. The indexed retention times (iRT-values) for these peptides are listed in Table 1. The data was analyzed and refined in Skyline [6] and the generated MSstats input file (Table 2) was processed with MSstats 3.1.4 [7]. A global standard normalization was used for the data with two endogenous peptides (SNLDSNHIIYR and SEELLGAASNR) of the probable DNA-directed RNA polymerase omega subunit (*ssl2982*). The data was processed with MSstats by using restricted scope of conclusions to the three replicates. The experimental design was based on MSstats implemented Group Comparison tool. The statistical information on the expression level changes of all quantified proteins can be found from Table 3. The entire SRM data can be viewed as a Skyline document in Panorama Public via following link: https://panoramaweb.org/labkey/SufR. The raw MS-data (.raw -files) and the transition list (.csv –file) can be accessed in the Peptide Atlas SRM Experiment Library (PASSEL) with an identifier PASS00765 (http://www.peptideatlas.org/PASS/PASS00765).

## Appendix A Supplementary material

Supplementary material **Table 1.** iRT values of the peptides which were not described in [5].

Supplementary material **Table 2**. MSstats input file.

Supplementary material **Table 3**. Statistical information on the expression level changes of all quantified proteins under all tested conditions.
